# Fatal *Paraclostridium sordellii* Infection: Post-Mortem Assessment and Review of the Literature

**DOI:** 10.3390/pathogens14070703

**Published:** 2025-07-16

**Authors:** Martina Focardi, Simone Faccioli, Beatrice Defraia, Rossella Grifoni, Ilenia Bianchi, Fabio Vaiano, Luca Novelli, Nunziata Ciccone, Emanuele Capasso, Francesca Malentacchi, Vilma Pinchi, Gian Maria Rossolini

**Affiliations:** 1 Forensic Medical Sciences, Department of Health Science, University of Florence, 50100 Florence, Italy; martina.focardi@unifi.it (M.F.); simone.faccioli@unifi.it (S.F.); rossella.grifoni@unifi.it (R.G.); ilenia.bianchi@unifi.it (I.B.); fabio.vaiano@unifi.it (F.V.); vilma.pinchi@unifi.it (V.P.); 2 Pathological Anatomy Unit, Careggi University Hospital, 50100 Florence, Italy; novellil@aou-careggi.toscana.it; 3 Microbiology and Virology Unit, Careggi University Hospital, 50100 Florence, Italy; nunziata.ciccone@unifi.it (N.C.); malentacchif@aou-careggi.toscana.it (F.M.); rossolini.gianmaria@unifi.it (G.M.R.); 4 Department of Advanced Biomedical Sciences—Legal Medicine Section, University of Naples “Federico II”, 80100 Naples, Italy; emanuele.capasso@unina.it; 5 Department of Experimental and Clinical Medicine, University of Florence, 50100 Florence, Italy

**Keywords:** *Clostridium sordellii*, *Paeniclostridium sordellii*, *Paraclostridium sordellii*, post-mortem microbiological investigations

## Abstract

*Clostridium sordellii*, which has recently been reclassified as *Paeniclostridium sordellii* and subsequently as *Paraclostridium sordellii*, is a rare human pathogen linked to infections of high morbidity and mortality, often presenting as fulminant toxic shock syndrome. Although most documented cases involve individuals with pre-existing health issues, such as immunosuppression and cancer, or those who have undergone specific gynecological procedures, there are few instances reported in otherwise healthy individuals. In this report, we present a case of fatality associated with *P. sordellii* infection in a young individual with a history of drug abuse, following post-mortem examinations. Additionally, we provide an updated review of the latest literature on this topic.

## 1. Introduction

*Clostridium sordellii* (recently renamed *Paeniclostridium sordellii* [1] and then *Paraclostridium sordellii* [2]) is an infrequent human pathogen associated with infections of high morbidity and mortality, possibly presenting as fulminant toxic shock syndrome. While most cases have been described in subjects with underlying conditions, including immunodepression [3] and oncological disease [4,5,6,7,8,9,10], or after specific gynecological procedures [11,12,13,14,15,16,17,18,19], few cases were reported among previously healthy subjects, mostly related to recent injury [20,21,22,23] or acute omphalitis in newborns [24,25,26,27,28]. A small number of cases is related to injection drug use [10,29,30,31], especially black tar heroin [32,33]. The largest review was performed by Aldape et al. in 2006 [34].

Here, we report on a case of death associated with *P. sordellii* infection in a young drug abuser, diagnosed by post-mortem microbiological investigations.

## 2. Case Presentation

Early in the morning on a cold winter’s day, a forty-year-old woman was discovered in a public park attended by drug users and other related individuals. She was warmly dressed due to the cold temperature, estimated between 9.1 and 11.7 degrees Celsius according to the weather service. She was found unconscious and unresponsive in her makeshift shelter by a groundskeeper. Medical assistance was called, and the first physician to arrive witnessed the woman’s death without attempting any resuscitative maneuvers. The physician noted that the body showed abundant hypostasis, consistent with the described position (the body was faced down). No traumatic lesions or venipunctures were detected. The woman tested positive for drug abuse on her medical history, which included intravenous heroin use dating back to her youth and, more recently, smoking crack cocaine.

Despite multiple hospitalizations and support from specific local centers in the rehabilitation community, all attempts at rehabilitation failed. Although the woman had stopped using needles, she continued to smoke crack, as recently documented. In the days preceding the discovery of her body, she showed no signs of illness and had not reported feeling unwell (the last contact with the rehab community occurred less than 24 h before her death).

The body was taken to the local Forensic Medicine Institute and stored (in supine position) in a mortuary refrigerator set to a constant temperature of 0–4 degrees Celsius until the autopsy, which was performed 72 h after the discovery.

**Autopsy findings**

An autopsy was conducted on the body, including a thorough external and internal post-mortem examination. During the external examination, the pathologist noted complete hypostasis migration, rigor mortis fully set in—although easily reversible—and a small area of greenish discoloration, measuring approximately 3 × 2 cm, in the upper left abdomen.

No traumatic injuries were found, but a reddish fluid was seen leaking from the nostrils. There were no venipunctures.

Upon dissecting the head, the pathologist found a moderately edematous brain with consistent texture. The abdominal and thoracic organs appeared deteriorated, showing changes in color and texture and smelled sickeningly sweet odor. The lungs were congested and edematous, the heart had no significant abnormalities, and the liver was enlarged and structurally altered, with microvesicular steatosis and fibrosis and steatohepatitis. The intestines were distended with gas, but no mucosal changes were detected.

During the autopsy, organ samples were taken for histological examination, biological fluids were collected for toxicological analysis, and samples and swabs were obtained for microbiological testing as per the Forensic Unit’s internal protocol.

**Histological findings**

**Figure 1:**
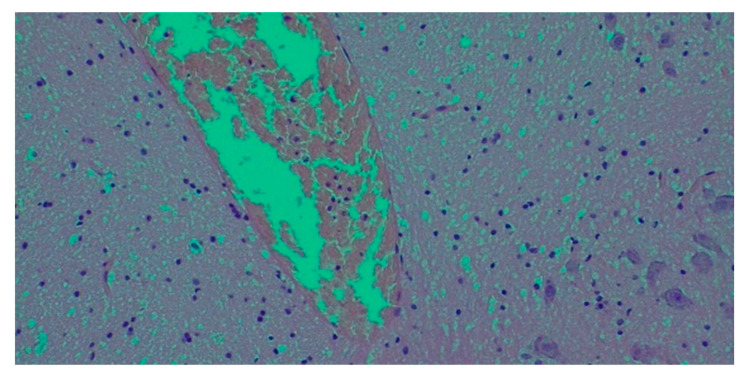
Brain oedema and congestion.

Multiple organ samples were taken from the lungs, brain, heart, kidneys, spleen, pancreas, and liver. They were included in paraffin and cut with a microtome into micrometric slides. Hematoxylin and eosin staining was performed, and specimens were observed under a microscope (Figure 1).

**Figure 2:**
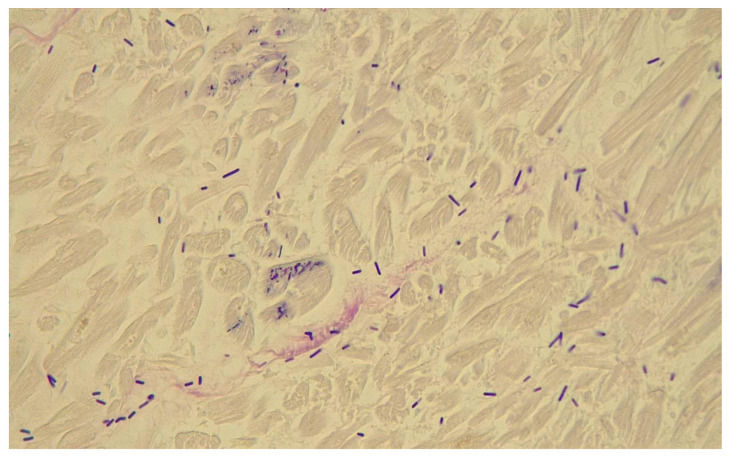
Gram staining in heart.

Histological examination revealed multi-visceral congestion, brain edema, lung edema, areas of cardiac necrosis with edema, and hepatic steatosis. In all parenchymas, congestion and the presence of lymphocytic and granulocytic infiltrates were found. Copious intravascular granulocytes and a lesser number of lymphocytes were also observed at the intravascular site. Gram-positive bacilli were widespread in the heart, lung, and kidney (Gram staining) (Figure 2). Immunohistochemical testing of tissues was conducted to disclose the inflammatory response and leukocytes cells. In particular, antibodies for CD15 (MMA) ^®^ and CD68 (PG-M1) ^®^ were obtained from Diagnostic BioSystems (DBS) (Pleasanton, CA, USA).

## 3. Results

**Post-mortem microbiological investigations**

Microbiological cultures were carried out on specimens collected post-mortem, including blood, urine, rectal swabs, pharyngeal swabs, and samples from heart, brain, lung (right and left anterior upper lobe, right and left anterior lower lobe and right medial anterior lobe), kidneys (right and left), liver and spleen.

Blood, urine and mucosal swabs were taken during the external examination, by puncture of the femoral artery, suprapubic puncture, and direct swabbing, respectively. Tissue samples were taken during autopsy. Before sampling of blood and urine, the skin was carefully disinfected with betadine and sterile test vials and needles were used.

Blood was directly inoculated in 4 blood culture vials for aerobic and anaerobic cultures (BactAlert system, Marcy l’Etoile, France, bioMérieux). Urine was collected in a sterile tube and then transferred to a urine Sponge^TM^ (Copan, Murrieta, CA, USA) according to the manufacturer’s instructions. FecalSwab^TM^ and UTM^TM^ transport media (Copan, Murrieta, CA, USA) were used for rectal and pharyngeal swabs, respectively. Other samples (1–2 cm^3^) were collected in sterile tubes containing minimal amount (0.5 mL) of sterile normal saline to preserve from drying out. All specimens were transferred to the laboratory and processed within 1 h. Culture media, incubation conditions and incubation times are reported in Table S1.

Bacterial identification was carried out by MALDI-ToF mass spectrometry (MALDI Biotyper^®^ Bruker Daltonics, Bremen, Germany). Identification of *P. sordellii* was further confirmed by a whole-genome sequencing (WGS)-based analysis of one isolate, using the TYGS server [35]. Table 1 shows, for each sample, which bacteria were detected and any details regarding their growth.

A BLASTn analysis performed at the NCBI server (https://blast.ncbi.nlm.nih.gov/, accessed on 4 June 2025) with the genomic sequence of the *P. sordellii* isolate did not detect the genes encoding the lethal toxin (*tcsL*) or the hemorrhagic toxin (*tcsH*). However, the presence of *plC* (phospholipase C), *sdL* (sordellilysin), and *nanS* (sialidase) genes was confirmed, suggesting their potential involvement in the observed cytotoxicity [36].

**Toxicological findings**

Toxicological analyses were performed on biological samples (central and peripheral blood, urine, vitreous humor and bile) collected during the autopsy and rapidly stored at −20 °C. Cocaine, and its main metabolite (benzoylecgonine), methadone, and lorazepam were quantified as reported in Table 2. Blood alcohol content was negative.

## 4. Discussion

The case is worth reporting primarily because it was a case of sudden death that autopsy and histological examination ascribed to a fulminant shock syndrome, which the associated microbiological workup suggested to be caused by a *P. sordellii* infection. The patient was a known cocaine addict, although they smoked the drug rather than injecting it, which is more common in these cases of chronic drugs abusers. However, in this instance, there were no signs of injections, which was recently confirmed by the patient who openly admitted to smoking crack. This case also emphasizes the importance of post-mortem microbiological samplings in cases of sudden death or death without a witness.

*Clostridium sordellii*, first described by Alfredo Sordelli [37] under the name “bacillus oedematis sporogenes” [38] and later categorized as *Paeniclostridium* [1] and then as *Paraclostridium* [2], is an anaerobic, Gram-positive, spore-forming rod implicated in acute and severe infections in both humans and animals [39,40,41]. Like other clostridia, *P. sordellii* is commonly found in soil but can also be present in a small percentage of the population in the vagina or rectum [42], although this percentage may vary between populations [43].

While non-pathogenic strains of *P. sordellii* exist, there are others that produce two unique toxins that are significant virulence factors [44]: the lethal toxin (TcsL) and the hemorrhagic toxin (TcsH), both of which interfere with the Rho pathway [45] and bear similarities to toxins produced by *Clostridioides difficile* [46,47]. *P. sordellii* also produces other exotoxins whose role in pathogenesis has not been extensively studied [34].

The lethal toxin was shown to induce a massive increase in vascular permeability in mice [48]. In humans, it causes a peculiar toxic shock syndrome strictly related to capillary leaking. This syndrome is characterized by diffuse edema, unresponsive to treatment hypotension with hemoconcentration, sometimes gastrointestinal or nonspecific symptoms, massive leukocytosis (frequently more than 50,000 cells/microL, a value so high it is called a “leukemoid reaction” [49]), and absence of fever, due to the specific anti-inflammatory properties of TcsL [50].

*P. sordellii* may also cause a less specific clinical syndrome: myonecrosis with gas gangrene that shares many features with other clostridial infections [51]. Like many other pathogenic bacteria, it can also cause a less common localized infection without any remarkable traits that may eventually evolve into sepsis.

Infections caused by *P. sordellii* exhibit two typical characteristics: from a clinical perspective, the rapid onset of tissue necrosis and shock leading to multi-organ failure; from a pathophysiological mechanism perspective, there is a generalized capillary leak and a massive increase in circulating leukocytes, defined as a “leukemoid reaction.”

Each clinical syndrome seems to be more closely related to a specific mode of infection: while toxic shock syndrome is predominant in infections related to obstetrics and gynecological procedures, myonecrosis seems to be prevalent in post-traumatic and drug-related infections, as well as in subjects with immunosuppression. Localized infections do not show any remarkable pattern. The largest review reported by Aldape et al. [34] highlighted that out of a total of 45 cases, the majority were related to gynecological procedures, with 11% involving medically induced abortions, 0.4% related to spontaneous abortions, and 18% concerning normal deliveries. In all three mentioned categories, the observed mortality rate was 100%. Conversely, 22% of the cases were linked to the use of injectable drugs, and in this category, a significantly lower mortality rate of 50% was observed. A similar mortality rate of 53% was noted for infections resulting from trauma or surgical procedures, which accounted for 43% of the sample. Considering the sample as a whole, the mortality rate was found to be 69% (31 subjects out of 45), with approximately 85% of deaths occurring within less than a week (2–6 days) and in the presence of a leukemoid reaction. Overall, these data show that infections caused by *P. sordellii*, although rare, present extremely high mortality rates, approaching 100% in certain patient categories; therefore, it is essential to maintain a high level of awareness regarding this pathogen [52,53]. Table 3 provides a list from the case reports and case series identified in the literature and some summary notes regarding the content of each.

The authors present here a case of death in a female drug addict who was found dead in a park. The autopsy and subsequent histopathological examinations revealed findings consistent with acute heart failure due to sepsis syndrome, including morphonuclear lymphocyte infiltration intravascular in the heart and lungs with granulocytes, as well as the presence of Gram-positive bacilli in the same organs. Microbiological workup performed on autopsy specimens revealed the presence of *P. sordellii* in two separate blood samples, which was considered highly suggestive, in combination with the patient’s history and the anatomical and histopathological findings, with a case of fulminant shock syndrome associated with *P. sordellii* infection.

However, it should be noted that the blood cultures were also positive for various species of coagulase negative staphylococci (which are common skin colonizers), which might reflect the occurrence of contamination upon sampling, and that one vial was also positive for *Enterococcus faecalis* and *Escherichia coli*, which are normally members of the intestinal microbiota. The latter finding could reflect the fact that autopsy was performed 72 h after time of death, i.e., when post-mortem changes might have led to the permeabilization of the intestinal wall and subsequent migration of gastrointestinal bacteria to inner organs and blood. Therefore, we cannot exclude that the occurrence of *P. sordellii* in the blood could have been the result of a post-mortem scenario.

Moreover, in our case, numerous Gram-positive bacilli were observed in the heart by microscopy, but upon cultivation, a Gram-negative rod (namely a Citrobacter freundii) was detected. The latter finding could reflect the fact that culture conditions used for tissue specimens were different from those uses for blood specimens, and might have been no permissive for growth of *P. sordellii*.

Toxicological analysis revealed consumption of lorazepam, methadone, and cocaine at “therapeutic/normal” levels. Bile concentrations suggested recent consumption of cocaine and methadone, confirming a history of cocaine abuse and therapeutic use of opioids. None of the substances played a significant role in the cause of death.

The forensic diagnosis and conclusion are consistent with the information available from what is reported in the literature regarding findings from rare cases. *P. sordellii* infections are characterized by the absence or only mild local inflammatory response. Experimental infection with *P. sordellii* induces edema, muscle fiber disruption, tissue necrosis, and significant increase in white blood cells, specifically neutrophils, in vessels of all tissue specimens.

The infection can occur in subjects who use drugs, especially injectable ones, as in this case. Regardless of the cause of infection, *P. sordellii* cases are often apyrexial and develop a profound leukemoid reaction, as witnessed here. The case involves a sudden death suspected to be caused by an overdose, occurring without any prodromal symptoms or signs of infection in the 24 h leading up to death, linked to *P. sordellii*, a pathology that is often lethal but infrequently encountered.

This case highlights the importance of microbiological testing in cases of sudden and/or unwitnessed deaths. Despite the difficulties related to interpreting microbiological results, which represent a limitation of our findings, they underscore the importance of integrating these results with macroscopic investigations during the autopsy and histopathological results can help in discriminating between microbiological positivity from post-mortem bacterial translocation or contamination and positivity from local or systemic infection contracted in the pre-mortem period.

## 5. Conclusions

The case presented highlights the significance of conducting comprehensive microbiological investigations in cases of sudden death, even in the absence of clear signs of an active infection. It is important to carefully evaluate the results of these tests, as several factors can affect them. Therefore, it is crucial to combine microbiological findings with a thorough examination that takes into account both the macroscopic and microscopic evidence seen during the autopsy.

## Figures and Tables

**Table 1:** Each sample, which bacteria were detected and any details regarding their growth.

Biospecimen	Microbes	Note
Heart	*Citrobacter freundii*	Obtained after broth enrichment
Brain	*Klebsiella ornithinolytica*	Rare colonies
Liver	*Lactobacillus rhamnosus*	Obtained after broth enrichment
Spleen	*Staphylococcus epidermidis*	Obtained after broth enrichment
	*Lactobacillus* spp.	
Right kidney	*Enterococcus gallinarum*	Rare colonies * Obtained after broth enrichment
	*Escherichia coli* *	
	*Bacterioides fragilis* *	
Left kidney	*Lactobacillus rhamnosus*	Obtained after broth enrichment
Lung: right anterior upper lobe	*Veillonella parvula*	Obtained after broth enrichment
Lung: right anterior medial lobe	*Lactobacillus rhamnosus*	Obtained after broth enrichment
Lung: right anterior lower lobe	Negative	
Lung: left anterior upper lobe	*Lactobacillus rhamnosus*	Discrete number of colonies * Obtained after broth enrichment
	*Veillonella parvula*	
	*Enterococcus faecalis* *	
Lung: left anterior lower lobe	*Enteroccoccus faecalis*	Obtained after broth enrichment
Blood 1st sample	*Staphyloccoccus epidermidis*	§ obtained from anaerobic culture
	*Staphylococcus lugdunensis*	
	*Enterococcus faecalis*	
	*Escherichia coli*	
	*Paraclostridium sordellii* §	
Blood 2nd sample	*Staphylococcus epidermidis*	§ obtained from anaerobic culture
	*Staphylococcus hominis*	
	*Paraclostridium sordellii* §	
Urine	Negative	
Rectal swab	Negative	
Nasopharyngeal swab SARS-CoV2	Negative	

* = Temperature of 35 °C (±1 °C) was used for incubation; § aerobic atmosphere enriched with 5% CO_2_ was used for incubation of CHOC plates; anaerobic atmosphere was obtained in dedicated sealed boxes using “Anaerogen^TM^ Atmosphere generation system (ThermoScientific) pad, or in dedicated incubator for blood cultures (BactAlert system, Marcy l’Etoile, France, bioMérieux)”.

**Table 2:** Cocaine, benzoylecgonine, methadone, and lorazepam concentrations in biological samples.

Compound	Concentrations (ng/mL)				
	Central Blood	Peripheral Blood	Urine	Vitreous Humor	Bile
Cocaine	3329	2197	2962	337	35,847
Benzoylecgonine	61	101	2120	125	-
Methadone	234	515	738	132	752
Lorazepam	12	11	-	<LOQ *	31

* LOQ: limit of quantification (1 ng/mL).

**Table 3:** List from the case reports and case series identified in the literature.

Authors	N° of Cases *	Subject Age and Sex	Type of Case	Fatal Outcome?	Autopsy Performed?	How Was *C.s.* Diagnosed?	Toxin Presence Verified?
Browdie et al., 1975 [20]	1	23 years M	Traumatic injury	yes	yes	Cultural from wound	no
Bogdan et al., 1976 [24]	1	13 days F	Omphalitis	yes	yes	Cultural from blood and fluid from the site of infection	no
Gormley et al., 1977 [25]	1	4 days F	Omphalitis	yes	yes	Cultural from fluid from the site of infection	no
Thys et al., 1980 [54]	1	18 years M	Other	yes **	yes	Isolated from muscle and blood sample	no
Barnes et al., 1987 [55]	1	61 years M	Other	no	--	Cultural from blood samples and pleural fluid	no
Hogan et al., 1989 [56]	1	39 years F	Gynecological/obstetrician	yes	yes	Cultural from an exudate of myometrium acquired at autopsy	no
McGregor et al., 1989 [12]	3	28 years F	Gynecological/obstetrician	yes	yes	Cultural from “clot” from vagina	no
		23 years F	Gynecological/obstetrician	yes	no	Cultural from cervical lesion	no
		23 years F	Gynecological/obstetrician	yes	yes	Cultural from intraoperative and autopsy samples	yes
Grimwood et al., 1990 [21]	1	4 years F	Traumatic injury	no	--	Cultural from necrotic tissue from the site of infection	yes
Kosloske et al., 1991 [26]	1	9 days F	Omphalitis	no	--	Cultural from exudate and tissues from site of infection	no
Buchman et al., 1991 [57]	1	95 years F	Other	no ***	no	Cultural from pleural fluid	no
Spera J, et al., 1992 [58]	1	37 years M	Drug related	no	--	Cultural from blood samples	no
Adamkiewicz et al., 1993 [27]	1	17 days F	Omphalitis	yes	yes	Cultural from peritoneal fluid and umbilical tissue	yes
Mory et al., 1995 [3]	1	48 years F	Immunosuppressed/oncological patient	yes	yes	Cultural from blood samples and liver tissue acquired at autopsy	yes
Bitti et al., 1996 [13]	1	29 years F	Gynecological/obstetrician	yes	no	Cultural from blood	yes
Cunnife et al., 1996 [4]	1	55 years M	Immunosuppressed/oncological patient	yes	yes	Cultural from blood and stool samples	yes
Sosolik et al., 1996 [14]	1	24 years F	Gynecological/obstetrician	yes	yes	Cultural from resected necrotic tissue	no
Borer et al., 1999 [59]	1	73 years M	Immunosuppressed/oncological patient	yes	yes	Cultural from blood samples	no
Gredlein et al., 2000 [22]	1	37 years M	Traumatic injury	no	--	Cultural from intraoperative samples from site of infection	no
Rorbye et al., 2000 [15]	1	40 years F	Gynecological/obstetrician	yes	no	Cultural from discharge from site of infection	no
Abdulla et al., 2000 [5]	2	81 years F	Other	yes	yes	Cultural from blood samples	no
		12 years M	Immunosuppressed/oncological patient	no	--	Cultural from blood samples	no
Cobo et al., 2001 [60]	1	17 years F	Other	no	--	Cultural from a corneal ulcer	no
CDC* 2001 [61]	1	23 years M	Other	yes	yes	Cultural from blood	no
Bangsberg et al., 2002 [32]	2	28 years F	Drug related	yes	no	Cultural from surgical wound	no
		52 years M	Drug related	no	--	Cultural from intraoperative sample of tissues from site of infection	no
Sinave et al., 2002 [16]	1	26 years F	Gynecological/obstetrician	yes	yes	Cultural from endometrial biopsy	no
Lorea et al., 2004 [62]	1	49 years M	Other	no	--	Cultural from perioperative samples	no
Zink et al., 2004 [63]	1	33 years M	Other	no	--	Cultural from vitreous sample	no
Wiebe et al., 2004 [64]	1	27 years F	Gynecological/obstetrician	yes	yes	Cultural from endometrial biopsy and perioperative samples	no
Fischer et al., 2005 [11]	4	18 years F	Gynecological/obstetrician	yes	yes	PCR assay from DNA extracted from uterine tissues fixed in formalin	yes
		21 years F	Gynecological/obstetrician	yes	yes	PCR assay from DNA extracted from uterine tissues fixed in formalin	yes
		22 years F	Gynecological/obstetrician	yes	yes	PCR assay from DNA extracted from uterine tissues fixed in formalin	yes
		34 years F	Gynecological/obstetrician	yes	yes	PCR assay from DNA extracted from uterine tissues fixed in formalin	yes
Brett et al., 2005 [29]	1	unknown M	Drug related	yes	no	not specified	no
Aldape et al., 2006 [34]	2	4 years M	Traumatic injury	yes	yes	Cultural from intraoperative samples from site of infection	no
		21 years F	Gynecological/obstetrician	yes	yes	Cultural from intraoperative samples from site of infection	no
Elsayed et al., 2006 [6]	1	61 years F	Immunosuppressed/oncological patient	yes	yes	Cultural from blood samples	yes
Cohen et al., 2007 [65]	2	25 years F	Gynecological/obstetrician	no	--	Cultural from blood and cervical samples	yes
		18 years F	Gynecological/obstetrician	yes	yes	Immunohistochemical and PCR assay on uterine tissue	yes
Matten et al., 2009 [7]	1	59 years M	Immunosuppressed/oncological patient	no	--	Cultural from blood samples and from pleural and liver drainage	yes
Foroulis et al., 2009 [66]	1	56 years M	Other	no	--	PCR analysis from pleural fluid	yes
Ho et al., 2009 [67]	2	32 years F	Gynecological/obstetrician	yes	yes	Immunohistochemical and PCR assay on fixed tissue	yes
		40 years F	Gynecological/obstetrician	yes	yes	Immunohistochemical and PCR assay on fixed tissue	yes
Valour et al., 2010 [68]	1	31 years F	Traumatic injury	no	--	PCR analysis on DNA from infected brain tissue	yes
Meites et al., 2010 [69]	2	29 years F	Gynecological/obstetrician	yes	no	PCR assay from infected tissue	no
		21 years F	Gynecological/obstetrician	yes	no	PCR assay from infected tissue	no
Chaudhry et al., 2011 [70]	1	8 months M	Other	no	--	Cultural and PCR assay from intraoperative samples	yes
Walk et al., 2011 [71]	1	81 years F	Other	no	--	Cultures from blood samples and PCR assay on the cultured strains	yes
Smith et al., 2013 [8]	1	37 years F	Immunosuppressed/oncological patient	no	--	Cultural from samples from site of infection	no
Marinis et al., 2013 [30]	1	25 years F	Drug related	no	--	Cultural from intraoperative samples from site of infection	no
Bouvet et al., 2015 [23]	1	78 years M	Traumatic injury	not specified	no	Cultural from blood and PCR assay on subculture	yes
Guzzetta et al., 2016 [72]	1	33 years F	Gynecological/obstetrician	yes	yes	Cultural from episiotomy site and blood samples	no
Rellinger et al., 2016 [28]	1	8 days M	Omphalitis	yes	yes	Cultural from intraoperative samples of tissues from site of infection	no
Bonnecaze et al., 2016 [9]	1	67 years F	Immunosuppressed/oncological patient	no	--	Cultural from blood samples	no
Gray et al., 2018 [73]	1	61 years M	Traumatic injury	not specified	--	Cultural from wound site	no
Mattson et al., 2018 [18]	1	45 years F	Gynecological/obstetrician	no	--	Cultural from intraoperative sample from site of infection	no
Elkubuli et al., 2018 [19]	1	31 years F	Gynecological/obstetrician	no	--	Immunohistochemical and PCR assay on intraoperative samples	no
Boyanton et al., 2019 [17]	1	29 years F	Gynecological/obstetrician	yes	no	Cultural from ascites samples	no
Silva et al., 2020 [31]	1	37 years F	Drug related	yes	no	Cultural from intraoperative samples from site of infection	no
Chaudhry et al., 2021 [74]	1	28 years F	Other	no	--	Cultural from intraoperative samples from site of infection	yes
Varley et al., 2022 [10]	1	63 years M	Immunosuppressed/oncological patient	no	--	Cultural from blood samples	yes
Milano et al., 2023 [75]	4	34 years M	Lupus/drug related	yes	no	Cultural from intraoperative samples from site of infection	yes
		41 years M	Drug related	no	--	Cultural from intraoperative samples from site of infection	yes
		42 years M	Morbid obesity/drug related	yes	yes	Cultural from abdominal wall tissue	yes
		47 years M	Drug related	yes	no	Cultural from intraoperative samples from site of infection	yes
Jacques et al., 2024 [76]	2	30 years F	42 weeks gestation	yes	yes	Uterine and placental cultures	yes
						Cultural from blood samples	no
		Adolescence F	Medication abortion	yes	yes	Endometrial culture	yes
Kurth et al., 2024 [77]	15	30	Drug related	no	--	Cultural from intraoperative samples from site of infection	yes
		69	Drug related	yes	no	Cultural from intraoperative samples from site of infection	yes
		33	Drug related	no	--	Cultural from intraoperative samples from site of infection	yes
		68	/	no	--	Cultural from intraoperative samples from site of infection	yes
		58	/	no	--	Cultural from intraoperative samples from site of infection	yes
		76	/	no	--	Cultural from intraoperative samples from site of infection	yes
		50	/	no	--	Cultural from intraoperative samples from site of infection	yes
		53	Drug related	no	--	Cultural from intraoperative samples from site of infection	yes
		64	/	no	--	Cultural from blood samples	yes
		42	Drug related	yes	no	Cultural from intraoperative samples from site of infection	yes
		54	/	no	--	Cultural from blood samples	yes
		60	Drug related	no	--	Cultural from intraoperative samples from site of infection	yes
		47	Drug related	yes	no	Cultural from intraoperative samples from site of infection	yes
		57	Drug related	yes	no	Cultural from intraoperative samples from site of infection	yes
		80	/	yes	no	Cultural from blood samples	yes

* In some cases, death reports due to other *Clostridia* were presented but had not been counted in the table. ** Brain death of the subject had been already declared; the infection led to cardiac arrest. *** Patient survived the infection but died of unknown causes after a prolonged hospitalization; / Not drug related.

## Data Availability

The original contributions presented in this study are included in the article/Supplementary Material. Further inquiries can be directed to the corresponding author.

## Supplementary Materials

The following supporting information can be downloaded at https://www.mdpi.com/article/10.3390/pathogens14070703/s1, Table S1: Culture media and conditions used for microbiological analysis of autoptic specimens.

## References

[1] Sasi Jyothsna T.S., Tushar L., Sasikala C., Ramana C.V. (2016). *Paraclostridium benzoelyticum* gen. nov., sp. nov., isolated from marine sediment and reclassification of *Clostridium bifermentans* as *Paraclostridium bifermentans* comb. nov. Proposal of a new genus *Paeniclostridium* gen. nov. to accommodate *Clostridium sordellii* and *Clostridium ghonii*. Int. J. Syst. Evol. Microbiol..

[2] Bello S., McQuay S., Rudra B., Gupta R.S. (2024). Robust demarcation of the family Peptostreptococcaceae and its main genera based on phylogenomic studies and taxon-specific molecular markers. Int. J. Syst. Evol. Microbiol..

[3] Mory F., Lozniewski A., Guirlet M.N., Guidat D., Bresler L., Weber M., Boissel P. (1995). Severe sepsis Caused by *Clostridium sordellii* Following Liver Biopsy in a Liver Transplant Recipient. Clin. Infect. Dis..

[4] Cunniffe J.G. (1996). *Clostridium sordellii* bacteraemia. J. Infect..

[5] Abdulla A., Yee L. (2000). The clinical spectrum of *Clostridium sordellii* bacteraemia: Two case reports and a review of the literature. J. Clin. Pathol..

[6] Elsayed S., Zhang K. (2006). Positive *Clostridium difficile* Stool Assay in a Patient with Fatal *C. sordellii* Infection. N. Engl. J. Med..

[7] Matten J., Buechner V., Schwarz R. (2009). A Rare Case of *Clostridium sordellii* Bacteremia in an Immunocompromised Patient. Infection.

[8] Smith C., Goslin B. (2013). *Clostridium sordellii* Surgical Site Infection after Breast Mass Excision: Case Report. Surg. Infect..

[9] Bonnecaze A.K., Stephens S.E.E., Miller P.J. (2016). Non-lethal *Clostridium sordellii* bacteraemia in an immunocompromised patient with pleomorphic sarcoma. BMJ Case Rep..

[10] Varley C.D., Rogers L.M., Dixon B.R., Bernard S.C., Lacy D.B., Sulpizio E., Aronoff D.M., Townes J.M. (2022). Persistent bacteremia and psoas abscess caused by a lethal toxin-deficient *Paeniclostridium sordellii*. Anaerobe.

[11] Fischer M., Bhatnagar J., Guarner J., Reagan S., Hacker J.K., Van Meter S.H., Poukens V., Whiteman D.B., Iton A., Cheung M. (2005). Fatal Toxic Shock Syndrome Associated with *Clostridium sordellii* after Medical Abortion. N. Engl. J. Med..

[12] McGregor J.A., Soper D.E., Lovell G., Todd J.K. (1989). Maternal deaths associated with *Clostridium sordellii* infection. Am. J. Obstet. Gynecol..

[13] Bitti A., Mastrantonio P., Spigaglia P., Urru G., Spano A.I., Moretti G., Cherchi G.B. (1996). A fatal postpartum *Clostridium sordellii* associated toxic shock syndrome. J. Clin. Pathol..

[14] Sosolik R.C., Savage B.A., Vaccarello L. (1996). *Clostridium sordellii* Toxic Shock Syndrome: A Case Report and Review of the Literature. Infect. Dis. Obstet. Gynecol..

[15] Rørbye C., Petersen I.S., Nilas L. (2000). Postpartum *Clostridium sordellii* infection associated with fatal toxic shock syndrome. Acta Obstet. Gynecol. Scand..

[16] Sinave C., Le Templier G., Blouin D., Léveillé F., Deland É. (2002). Toxic Shock Syndrome Due to *Clostridium sordellii:* A Dramatic Postpartum and Postabortion Disease. Clin. Infect. Dis..

[17] Boyanton Jr B.L., Hanna M.M., Hafez-Khayyata S., Robinson-Dunn B. (2020). Fatal Postpartum Infection. Infect. Dis. Clin. Pract..

[18] Mattson J.N., Hardy-Fairbanks A.J. (2018). *Clostridium sordelli* Toxic Shock After Endometrial Ablation: Review of Gynecologic Cases. J. Gynecol. Surg..

[19] Elkbuli A., Diaz B., Ehrhardt J.D., Hai S., Kaufman S., McKenney M., Boneva D. (2018). Survival from Clostridium toxic shock syndrome: Case report and review of the literature. Int. J. Surg. Case Rep..

[20] Browdie D.A., Davis J.H., Koplewitz M.J., Corday L., Leadbetter A.W. (1975). *Clostridium Sordelli* Infection. J. Trauma Acute Care Surg..

[21] Grimwood K., Evans G.A., Govender S.T., Woods D.E. (1990). *Clostridium sordellii* infection and toxin neutralization. Pediatr. Infect. Dis. J..

[22] Gredlein C.M., Silverman M.L., Downey M.S. (2000). Polymicrobial Septic Arthritis Due to Clostridium Species: Case Report and Review. Clin. Infect. Dis..

[23] Bouvet P., Sautereau J., Le Coustumier A., Mory F., Bouchier C., Popoff M.R. (2015). Foot Infection by *Clostridium sordellii*: Case Report and Review of 15 Cases in France. J. Clin. Microbiol..

[24] Bogdan J.C., Rapkin R.H. (1976). Clostridia infection in the newborn. Pediatrics.

[25] Gormley D. (1977). Neonatal anaerobic (clostridial) cellulitis and omphalitis. Arch Dermatol..

[26] Kosloske A.M., Bartow S.A. (1991). Debridement of periumbilical necrotizing fasciitis: Importance of excision of the umbilical vessels and urachal remnant. J. Pediatr. Surg..

[27] Adamkiewicz T.V., Goodman D., Burke B., Lyerly D.M., Goswitz J., Ferrieri P. (1993). Neonatal *Clostridium sordellii* toxic omphalitis. Pediatr. Infect. Dis. J..

[28] Rellinger E.J., Craig B.T., Craig-Owens L.D., Pacheco M.C., Chung D.H., Danko M.E. (2016). *Clostridium sordellii* necrotizing omphalitis: A case report and literature review. J. Pediatr. Surg. Case Rep..

[29] Brett M.M., Hood J., Brazier J.S., Duerden B.I., Hahné S.J. (2005). Soft tissue infections caused by spore-forming bacteria in injecting drug users in the United Kingdom. Epidemiol. Infect..

[30] Marinis A., Voultsos M., Grivas P., Dikeakos P., Liarmakopoulos E., Paschalidis N., Rizos S. (2013). Vacuum-assisted therapy accelerates wound healing in necrotizing soft tissue infections: Our experience in two intravenous drug abuse patients. Infez. Med..

[31] Silva J., Henry R., Strickland M., Wang D., Matsushima K. (2021). Rapidly fatal necrotizing soft tissue infection due to *Clostridium sordellii* in an injection drug user. Am. J. Emerg. Med..

[32] Bangsberg D.R., Rosen J.I., Aragón T., Campbell A., Weir L., Perdreau-Remington F. (2002). Clostridial Myonecrosis Cluster Among Injection Drug Users. Arch. Intern. Med..

[33] Dunbar N.M., Harruff R.C. (2007). Necrotizing Fasciitis: Manifestations, Microbiology and Connection with Black Tar Heroin. J. Forensic Sci..

[34] Aldape M.J., Bryant A.E., Stevens D.L. (2006). *Clostridium sordellii* Infection: Epidemiology, Clinical Findings, and Current Perspectives on Diagnosis and Treatment. Clin. Infect. Dis..

[35] Meier-Kolthoff J.P., Göker M. (2019). TYGS is an automated high-throughput platform for state-of-the-art genome-based taxonomy. Nat. Commun..

[36] Awad M.M., Singleton J., Lyras D. (2016). The Sialidase NanS Enhances Non-TcsL Mediated Cytotoxicity of *Clostridium sordellii*. Toxins.

[37] Stoppani A., Sordelli D.O., Méndez B.S. (2001). Remembering a microbiologist: Alfredo Sordelli (1891–1967). Int. Microbiol..

[38] Hall I.C., Scott J.P. (1927). Bacillus Sordellii, a Cause of Malignant Edema in Man. J. Infect. Dis..

[39] Junior C.A.O., Silva R.O.S., Lobato F.C.F., Navarro M.A., Uzal F.A. (2020). Gas gangrene in mammals: A review. J. Vet. Diagn. Invest..

[40] Uzal F.A., Navarro M.A., Asin J., Henderson E.E. (2022). Clostridial Diseases of Horses: A Review. Vaccines.

[41] Gornatti-Churria C.D., Crispo M., Shivaprasad H.L., Uzal F.A. (2018). Gangrenous dermatitis in chickens and turkeys. J. Vet. Diagn. Invest..

[42] Chong E., Winikoff B., Charles D., Agnew K., Prentice J.L., Limbago B.M., Platais I., Louie K., Jones H.E., Shannon C. (2016). Vaginal and Rectal *Clostridium sordellii* and *Clostridium perfringens* Presence Among Women in the United States. Obstet. Gynecol..

[43] Ohashi Y., Fujisawa T. (2019). Analysis of Clostridium cluster XI bacteria in human feces. Biosci. Microbiota Food Health.

[44] Vidor C., Awad M., Lyras D. (2015). Antibiotic resistance, virulence factors and genetics of *Clostridium sordellii*. Res. Microbiol..

[45] Jank T., Aktories K. (2008). Structure and mode of action of clostridial glucosylating toxins: The ABCD model. Trends Microbiol..

[46] Green G.A., Schué V., Monteil H. (1995). Cloning and characterization of the cytotoxin L-encoding gene of *Clostridium sordellii: Homology* with clostridium difficile cytotoxin B. Gene.

[47] Sirigi Reddy A.R., Girinathan B.P., Zapotocny R., Govind R. (2013). Identification and Characterization of *Clostridium sordellii* Toxin Gene Regulator. J. Bacteriol..

[48] Geny B., Khun H., Fitting C., Zarantonelli L., Mazuet C., Cayet N., Szatanik M., Prevost M.C., Cavaillon J.M., Huerre M. (2007). *Clostridium sordellii* Lethal Toxin Kills Mice by Inducing a Major Increase in Lung Vascular Permeability. Am. J. Pathol..

[49] Aronoff D.M. (2013). *Clostridium novyi*, *sordellii*, and *tetani*: Mechanisms of disease. Anaerobe.

[50] Popoff M.R. (2018). *Clostridium difficile* and *Clostridium sordellii* toxins, proinflammatory versus anti-inflammatory response. Toxicon.

[51] Bryant A.E., Stevens D.L. (2010). Clostridial Myonecrosis: New Insights in Pathogenesis and Management. Curr. Infect. Dis. Rep..

[52] García M.G., Pérez-Cárceles M.D., Osuna E., Legaz I. (2020). Impact of the Human Microbiome in Forensic Sciences: A Systematic Review. Appl. Environ. Microbiol..

[53] Schulz M., Schmoldt A., Andresen-Streichert H., Iwersen-Bergmann S. (2020). Revisited: Therapeutic and toxic blood concentrations of more than 1100 drugs and other xenobiotics. Crit. Care.

[54] Thys J.P., Ectors P., Noel P. (1980). Non-traumatic clostridial myositis: An unusual feature of brain death. Postgrad. Med. J..

[55] Barnes P., Leedom J.M. (1987). Infective endocarditis due to *Clostridium sordellii*. Am. J. Med..

[56] Hogan S.F., Ireland K. (1989). Fatal acute spontaneous endometritis resulting from *Clostridium sordelli*. Am. J. Clin. Pathol..

[57] Buchman A.L., Ponsillo M., Nagami P.H. (1991). Empyema caused by *Clostridium sordellii*, a rare form of pleuropulmonary disease. J. Infect..

[58] Spera R.V., Kaplan M.H., Allen S.L. (1992). *Clostridium sordellii* Bacteremia: Case Report and Review. Clin. Infect. Dis..

[59] Borer A., Gilad J., Sikuler E., Riesenberg K., Schlaeffer F., Buskila D. (1999). Fatal *Clostridium sordellii* ischio-rectal abscess with septicaemia complicating ultrasound-guided transrectal prostate biopsy. J. Infect..

[60] Cobo F., Aliaga L., Miranda C., de la Rosa M. (2001). *Clostridium sordelii* corneal ulcer. Infection.

[61] Centers for Disease Control and Prevention (CDC) (2001). Update: Unexplained deaths following knee surgery—Minnesota, 2001. MMWR Morb. Mortal Wkly. Rep..

[62] Lorea P., Baeten Y., Chahidi N., Franck D., Moermans J.P. (2004). A severe complication of muscle transfer: Clostridial myonecrosis. Ann. Chir. Plast. Esthet..

[63] Zink J.M., Singh-Parikshak R., Sugar A., Johnson M.W. (2004). *Clostridium sordellii* Endophthalmitis After Suture Removal from a Corneal Transplant. Cornea.

[64] Wiebe E., Guilbert E., Jacot F., Shannon C., Winikoff B. (2004). A Fatal Case of *Clostridium sordellii* Septic Shock Syndrome Associated with Medical Abortion. Obstet. Gynecol..

[65] Cohen A.L., Bhatnagar J., Reagan S., Zane S.B., D'Angeli M.A., Fischer M., Killgore G., Kwan-Gett T.S., Blossom D.B., Shieh W.J. (2007). Toxic shock associated with *Clostridium sordellii* and *Clostridium perfringens* after medical and spontaneous abortion. Obstet. Gynecol..

[66] Foroulis C.N., Gerogianni I., Kouritas V.K., Karestsi E., Klapsa D., Gourgoulianis K., Petinaki E. (2007). Direct detection of *Clostridium sordellii* in pleural fluid of a patient with pneumonic empyema by a broad-range 16S rRNA PCR. Scand. J. Infect. Dis..

[67] Ho C.S., Bhatnagar J., Cohen A.L., Hacker J.K., Zane S.B., Reagan S., Fischer M., Shieh W.J., Guarner J., Ahmad S. (2009). Undiagnosed cases of fatal Clostridium-associated toxic shock in Californian women of childbearing age. Am. J. Obstet. Gynecol..

[68] Valour F., Boisset S., Lebras L., Martha B., Boibieux A., Perpoint T., Chidiac C., Ferry T., Peyramond D. (2010). *Clostridium sordellii* Brain Abscess Diagnosed by 16S rRNA Gene Sequencing. J. Clin. Microbiol..

[69] Meites E., Zane S., Gould C. (2010). Fata l *Clostridium sordellii* Infections after Medical Abortions. N. Engl. J. Med..

[70] Chaudhry R., Verma N., Bahadur T., Chaudhary P., Sharma P., Sharma N. (2011). *Clostridium sordellii* as a Cause of Constrictive Pericarditis with Pyopericardium and Tamponade. J. Clin. Microbiol..

[71] Walk S.T., Jain R., Trivedi I., Grossman S., Newton D.W., Thelen T., Hao Y., Songer J.G., Carter G.P., Lyras D. (2011). Non-toxigenic *Clostridium sordellii*: Clinical and microbiological features of a case of cholangitis-associated bacteremia. Anaerobe.

[72] Guzzetta M., Williamson A., Duong S. (2016). *Clostridium Sordellii* as an Uncommon Cause of Fatal Toxic Shock Syndrome in a Postpartum 33-Year-Old Asian Woman, and the Need for Antepartum Screening for This Clostridia Species in the General Female Population. Lab. Med..

[73] Gray S.F., Dieudonne B.E. (2018). *Clostridium sordelli* causing malignant edema in a trauma patient: A case report and review of literature. Pan Afr. Med. J..

[74] Chaudhry R., Bahadur T., Sagar T., Agrawal S.K., Arif N., Choudhary S.K., Verma N. (2021). Infective Endocarditis Caused by *C. sordellii:* The First Case Report from India. J. Lab. Physicians.

[75] Milano V.R., Cabanilla M.G. (2023). Under the Skin: A Case Series of *Clostridium sordellii* Necrotizing Soft Tissue Infections in Patients Who Inject Drugs. Cureus.

[76] Jacques L., Kelly B., Soehl J., Wagar M., Rhoades J., Cowley E.S., Pryde P.G., Cutler A., Eschenbach D. (2024). Peripartum Uterine Clostridial Myonecrosis: A Report of Two Fatal Cases. WMJ.

[77] Kurth L., Johnston W., Black K., Doucet J., Weaver J. (2024). Mortality in a *Clostridium sordellii* Case Series. J. Surg. Res..

