# Supplementary material for: Fatal Paraclostridium sordellii Infection: Post-Mortem Assessment and Review of the Literature
Source: Pathogens. 2025 Jul 16;14(7):703. doi: 10.3390/pathogens14070703 (PMC12300805; doi:10.3390/pathogens14070703)
Supplement: Supplementary file 1 [file pathogens-14-00703-s001.zip › pathogens-3653972-supplementary.pdf]

Table S1. Culture media and conditions used for microbiological analysis of autoptic specimens.

Specimen	Type of culture	Incubation conditions (atmosphere§, temperature*)	Media°	Incubation times
**Biopsies**	Direct inoculum	Aerobic	CHOC and SDA	24h +24h
		Anaerobic	Schaedler	48h
	Enrichment	Aerobic	BHI	10 days
		Aerobic	THI	10 days
**Fecal swab**		Aerobic	CARB/OXA and Blood	16h
**Blood**	Enrichment	Aerobic	BactAlert aerobic medium	Until positivity reported by the system (max incubation time, 5 days)
		Anaerobic	BactAlert anaerobic medium	
**Positive aerobic blood culture**	Subculture	Aerobic	CHOC and SDA	24h
**Positive anaerobic blood culture**	Subculture	Anaerobic	Schaedler	24h
**Urine**		aerobic	CPSE	16h

Blood: Blood Agar Base plate

Acronym

§ aerobic atmosphere enriched with 5% CO_2 was used for incubation of CHOC plates; anaerobic atmosphere was obtained in dedicated sealed boxes using "Anaerogen™ Atmosphere generation system (ThermoScientific) pad, or in dedicated incubator for blood cultures (BactAlert System, bioMérieux).

* Temperature of 35°C (±1°C) was used for incubation.

CHOC, chocolate agar (Liofilchem); SDA, Sabouraud Dextrose Agar(Liofilchem); Schaedler, Schaedler Blood agar (bioMérieux); BHI, Brain heart infusion broth (Biolife); THI, Thioglycolate broth (Biolife); CARB/OXA, ChromID Carba SMART medium (bioMérieux); Blood, Blood agar (Liofilchem); CPSE, CHROMID-CPS ELITE medium (bioMérieux).
